# A Canadian survey of residency applicants’ and interviewers’ perceptions of the 2021 CaRMS R1 virtual interviews

**DOI:** 10.1186/s12909-023-04397-9

**Published:** 2023-05-30

**Authors:** Rosephine Del Fernandes, Nicole Relke, Eleftherios Soleas, Heather Braund, Clementine Janet Pui Man Lui, Boris Zevin

**Affiliations:** 1 School of Medicine, Queen’s University, Kingston, Canada; 2 Department of Medicine, University of Toronto, Toronto, Canada; 3 Office of Professional Development and Educational Scholarship, Faculty of Health Sciences, Queen’s University, Kingston, Canada; 4 Department of Medicine, Queen’s University, Kingston, Canada; 5 Department of Surgery, Queen’s University, Kingston General Hospital, 76 Stuart Street, Kingston, ON K7L 2V7 Canada

**Keywords:** CaRMS, Faculty interviewer, Medical applicant, R1 interviews, Residency Match, Residency Interviews, Residency applicants, Virtual Interviews

## Abstract

**Background:**

All Canadian Residency Matching Service (CaRMS) R1 interviews were conducted virtually for the first time in 2021. We explored the facilitators, barriers, and implications of the virtual interview process for the CaRMS R1 match and provide recommendations for improvement.

**Methods:**

We conducted a cross-sectional survey study of CaRMS R1 residency applicants and interviewers across Canada in 2021. Surveys were distributed by email to the interviewers, and by email, social media, or newsletter to the applicants. Inductive thematic analysis was used for open-ended items. Recommendations were provided as frequencies to demonstrate strength. Close-ended items were described and compared across groups using Chi-Square Fisher’s Exact tests.

**Results:**

A total of 127 applicants and 400 interviewers, including 127 program directors, responded to the survey. 193/380 (50.8%) interviewers and 90/118 (76.3%) applicants preferred virtual over in-person interview formats. Facilitators of the virtual interview format included cost and time savings, ease of scheduling, reduced environmental impact, greater equity, less stress, greater reach and participation, and safety. Barriers of the virtual interview format included reduced informal conversations, limited ability for applicants to explore programs at different locations, limited ability for programs to assess applicants’ interest, technological issues, concern for interview integrity, limited non-verbal communication, and reduced networking. The most helpful media for applicants to learn about residency programs were program websites, the CaRMS/AFMC websites, and recruitment videos. Additionally, panel interviews were preferred by applicants for their ability to showcase themselves and build connections with multiple interviewers. Respondents provided recommendations regarding: (1) dissemination of program information, (2) the use of technology, and (3) the virtual interview format.

**Conclusions:**

Perceptions of 2021 CaRMS R1 virtual interviews were favourable among applicants and interviewers. Recommendations from this study can help improve future iterations of virtual interviews.

**Supplementary Information:**

The online version contains supplementary material available at 10.1186/s12909-023-04397-9.

## Background

Historically, the performance of medical students on their residency interviews was used by the residency program selection committees to assess applicants’ fit for their program. Medical students rely on electives and residency interviews to choose a residency program that is the best fit for them [1, 2]. With the COVID-19 pandemic, postgraduate medical education programs across Canada cancelled all visiting electives starting March of 2020, limiting opportunities for medical students to gain in-person exposure and showcase their abilities and interests to programs outside of their home school [3].

The Canadian Resident Matching Service (CaRMS) is a national, independent, not-for-profit, fee-for-service organization that provides a centralized application and matching service for medical students applying to residency training programs across Canada. Approximately 3000 Canadian residency applicants apply yearly through this centralized program. Individual residency programs review applications, decide on the format and structure of the interview for their program, and conduct interviews directly with applicants who are offered an interview. For the first time in 2021, CaRMS changed its processes for matching applicants into residency programs, requiring all Canadian residency programs to conduct residency interviews virtually [2–5]. After the interviews, applicants and residency programs submit their preferred ranking of programs and candidates, respectively, who then undergo a match.

There are, on average, 50,000 Canadian residency interviews conducted each year [3], yet there is a paucity of studies exploring experiences of entry-level Canadian residency match (R1) applicants and interviewers with the virtual CaRMS interviews [6]. To date, studies have described fellowship applicants [4, 7–10], experiences from a single institution [7–9, 11], a single R1 specialty [2, 5, 12, 13], and applicants outside of Canada [9, 12–14]. Interviewers’ perspectives and preferences are mostly absent. A previous study by our group explored the perceptions and experiences of Canadian internal medicine residents and subspecialty medicine interviewers during the virtual interviews and found most in support of conducting interviews virtually [6]. Residents in Internal Medicine and final year medical students (applicants to the CaRMS R1 match) inherently have different opportunities to explore residency training programs as part of their training, which can impact their perspectives of the interview process. Additionally, our study has been adapted to explore the perspectives of interviewers within and outside of internal medicine and its subspecialties and of a larger cohort of residency applicants across all direct-entry residency programs. By identifying potential facilitators, barriers, and implications of the first iteration of virtual interviews for the 2021 CaRMS R1 match from the perspective of applicants and interviewers, we can begin to refine and improve the virtual interview process for the subsequent years and inform future decisions regarding the use of virtual interviews in the CaRMS R1 match.

The purpose of our study was to explore perceptions of applicants and interviewers regarding the preparation, facilitators, barriers, and implications of the first iteration of the virtual interview process for the 2021 CaRMS R1 match.

## Methods

### Setting and design

We conducted a web-based cross-sectional survey study of CaRMS R1 applicants and interviewers across Canada. We developed the survey from a literature review [15–17] and by modifying a previous survey administered to Canadian internal medicine subspecialty applicants by Relke et al. [6]. We pilot tested the survey with five program directors and five CaRMS R1 applicants at one Canadian university. Their feedback was used to revise the survey items to improve clarity and address additional areas of interest. The survey consisted of 30 questions for the CaRMS R1 applicants and 16 questions for interviewers with open-ended questions, checklist items, yes/no options, and 5-point Likert scales from “strongly disagree” to “strongly agree” (See the Additional file 1 for the full survey). The survey was created using the Qualtrics platform.

### Participant recruitment

We recruited residency program interviewers by emailing the program administrators of all 375 R1 programs listed on the CaRMS website for distribution to the interviewers within their respective program. All interviewers involved in the R1 virtual interviews were invited to participate, including program directors, faculty members, residents, allied health workers, and administrative support staff. We approached the medical school contact responsible for the class listserv of 14 English-speaking Canadian medical schools and asked them to distribute our survey to final-year medical students (CaRMS R1 applicants). We also used social media posts on Twitter, Facebook and medical schools’ newsletters to recruit applicants for our study. Duplicate entries were avoided using cookies to avoid two entries from the same computer.

### Survey distribution

Emails with the link to the survey and one email reminder were distributed after the rank-order deadline to the interviewers (April 5^th^ and April 12^th^, 2021) and to the CaRMS R1 applicants after they matched to their residency programs (April 29^th^, 2021 and June 8^th^, 2021) to ensure that participation in the survey would not affect residency match results. To further mitigate bias, surveys were kept anonymous, voluntary, and no personal information was collected. The link to the survey was open until June 30^th^, 2021.

### Outcomes measured

The primary outcome for our study was applicants’ and interviewers’ preferences regarding virtual and in-person CaRMS interview formats. Secondary outcomes were the reasons for choosing a preferred interview format, and perceived facilitators, barriers, and suggested improvements for the virtual interview format. We also explored applicants’ and interviewers’ experiences with technological issues, various interview formats, and different videoconferencing platforms.

### Analysis of the outcomes

#### Quantitative analysis

We present data as proportions for categorical data. We present rank lists in the order of increasing weighted averages across rank scores, with the mean of the top half rankings considered in a tie. We used Chi-square and Fisher’s exact tests for binomials to compare responses between applicants and interviewers, and by interviewer position. We included all survey responses in our analysis if the respondent completed questions beyond the demographic section. Each question was analyzed according to the number of respondents who answered that question. Quantitative analyses were conducted using SPSS version 27.

#### Qualitative analysis

We analyzed the qualitative data from open response questions using inductive thematic analysis [18]. One of the researchers (HB) was an external non-medical reviewer not involved in the initial protocol process to mitigate bias. As part of the coding process, two researchers (RDF and HB) collaboratively generated the initial codebook by coding 5% of applicant and interviewer responses to each question and then coding 10% independently. Intercoder agreement was found to be 95%. This agreement level was found by comparing each segment of text coded independently and calculating the number of times that the two researchers agreed versus disagreed generating an overall percentage. For the other 5%, researchers discussed the coded segments of text and made changes once agreement was reached. We used the consensus-built codebook for all remaining coding. After coding, similar codes were grouped together to form subthemes, and these were subsequently grouped together to form themes. These preliminary themes were discussed with the wider research team. After the intercoder reliability check, one researcher (RDF) analyzed the remaining qualitative data from open response questions. Data reached thematic saturation prior to the analyses of all responses. All data were coded past data saturation for representation across respondents. Qualitative analyses were conducted using Nvivo12.

To differentiate the strength of the recommendations in our study, we counted and displayed the themes describing recommendations as frequencies.

This study has been approved by the Queen’s University Health Sciences and Affiliated Teaching Hospitals Research Ethics Board (#6,030,219). All methods were carried out in accordance with relevant guidelines and regulations.

## Results

### Respondents’ characteristics

Five out of 14 English-speaking Canadian medical schools confirmed email distribution to their respective final year medical school classes. Eighty-six applicants responded to our survey from these five schools, with a response rate ranging from 5.0% to 19.9% per school. An additional three applicants responded through newsletter recruitments and 38 through social media recruitment, for a total of 127 applicants responding to our survey. A total of 400 interviewers from all 29 R1 disciplines responded to our survey, with 127 out of 410 program directors (31.0%) responding. Table 1 details the demographic information of the respondents, including their residency program or programs applied to, position, gender, and/or medical school attended.

**Table 1:** Respondent Demographic Information

Residency Program / Residency Program(s) Applied to	Interviewers, *N* (%)	Applications to each discipline, *N* (%)	Interviewer Position	Interviewers, *N* (%)
Anatomical pathology	14 (3.5)	2 (1.0)	Program Director	127 (31.8)
Anesthesiology	29 (7.2)	5 (2.4)	Faculty Member	152 (38.0)
Cardiac surgery	4 (1.0)	5 (2.4)	Resident	85 (21.3)
Dermatology	3 (0.8)	3 (1.5)	Other – Admin staff or Allied Health	36 (9)
Diagnostic Radiology	15 (3.8)	4 (2.0)	**Applicant Gender**	**Applicants,** ***N* (%)**
Emergency medicine	18 (4.5)	12 (5.9)	Man	45 (35.4)
Family medicine	48 (12.0)	76 (37.1)	Woman	81 (63.8)
General Pathology	3 (0.8)	2 (1.0)	Non-binary	1 (0.8)
General Surgery	18 (4.5)	11 (5.4)	**Medical School**	**Applicants,** ***N* (%)**
Hematological Pathology	6 (1.5)	0 (0)		
Internal Medicine	22 (5.5)	31 (15.1)	University of British Columbia	2 (1.6)
Medical Genetics and Genomics	8 (2.0)	2 (1.0)	University of Calgary	3 (2.4)
Medical Microbiology	6 (1.5)	1 (0.5)	University of Alberta	10 (7.9)
Neurology	12 (3.0)	1 (0.5)	University of Saskatchewan	5 (3.9)
Neuropathology	5 (1.3)	0 (0)	University of Manitoba	5 (3.9)
Neurosurgery	12 (3.0)	0 (0)	Western University	34 (26.8)
Nuclear Medicine	3 (0.8)	1 (0.5)	McMaster University	3 (2.4)
Obstetrics and gynecology	15 (3.8)	8 (3.9)	University of Toronto	3 (2.4)
Ophthalmology	17 (4.3)	1 (0.5)	Northern Ontario School of Medicine	10 (7.9)
Orthopedic surgery	12 (3.0)	2 (1.0)	Queen’s University	17 (13.4)
Otolaryngology – Head & Neck Surgery	10 (2.5)	2 (1.0)	University of Ottawa	1 (0.8)
Pediatrics	26 (6.5)	11 (5.4)	McGill University	6 (4.7)
Physical medicine and rehabilitation	11 (2.8)	2 (1.0)	Université de Montréal	0 (0)
Plastic surgery	5 (1.3)	5 (2.4)	Université de Sherbrooke	0 (0)
Psychiatry	31 (7.8)	8 (3.9)	Université Laval	4 (3.1)
Public health and preventive medicine	10 (2.5)	1 (0.5)	Dalhousie University	20 (15.7)
Radiation Oncology	26 (6.5)	1 (0.5)	Memorial University of Newfoundland	0 (0)
Urology	8 (2.0)	4 (2.0)	International medical graduates	2 (1.6)
Vascular Surgery	3 (0.8)	4 (2.4)	Undisclosed	2 (1.6)
**Total**	**400 (100)**^**a**^	**205 (100)**^**b**^		**127 (100)**^**c**^

^a^Total Interviewers

^b^Applications to each discipline. Individual applicants had the opportunity to submit multiple applications to different disciplines

^c^Total Applicants

A total of 119/127 (93.7%) applicants and 386/400 (96.5%) interviewers who responded went on to complete the survey. A total of 97/127 (76.4%) applicants and 300/400 (75.0%) interviewers provided qualitative comments for analysis. A total of 28/127 (22.0%) applicants reported having financial needs, 11/127 (8.7%) identified being from a remote or underserved community, and 11/127 (8.7%) identified with both characteristics.

Overall, 79/119 (66.4%) applicants and 156/386 (40.4%) interviewers rated the virtual CaRMS interview process as “good” or “excellent.” Applicants were significantly more likely than interviewers to prefer virtual over in-person CaRMS R1 interviews in the future (90/118 (76.3%) vs 193/380 (50.8%); *p* < 0.001). Among the interviewers, residents were less likely to prefer virtual over in-person CaRMS R1 interviews in the future (32/83, 38.6%, *p* = 0.013), compared to program directors (64/125, 51.2%), faculty (79/144, 54.9%), and others (18/28, 64.3%). Among the applicants, there were no significant differences for preferences for virtual or in-person interviews across the disciplines with 10 or more applicants (Family Medicine, Internal Medicine, Emergency Medicine, Pediatrics, and General Surgery) (*p* = 0.261).

### Applicants’ perspectives

#### Media for disseminating information about residency programs

The top four most helpful media ranked by applicants for providing information about residency programs were: (1) information on the CaRMS/AFMC website, (2) residency program websites, (3) online videos created by residency programs, and (4) virtual townhalls. After these, applicants ranked the following in decreasing order of helpfulness: (5) residency program social media presence, (6) emailed information from the programs, (7) informal discussions with residents and alumni outside of scheduled events, and (8) virtual social events offered to the candidates selected for the interview.

#### Strategies used by the applicants to prepare for the Virtual CaRMS Interviews

Most applicants optimized their physical space and conducted a technology trial run (114/125, 91.2%). Many applicants purchased new hardware (67/125**,** 53.6%), some moved to a location with reliable internet access (34/125, 27.2%) and upgraded their internet connection (16/125, 12.8%).

#### Virtual interview format

**Fig. 1:**
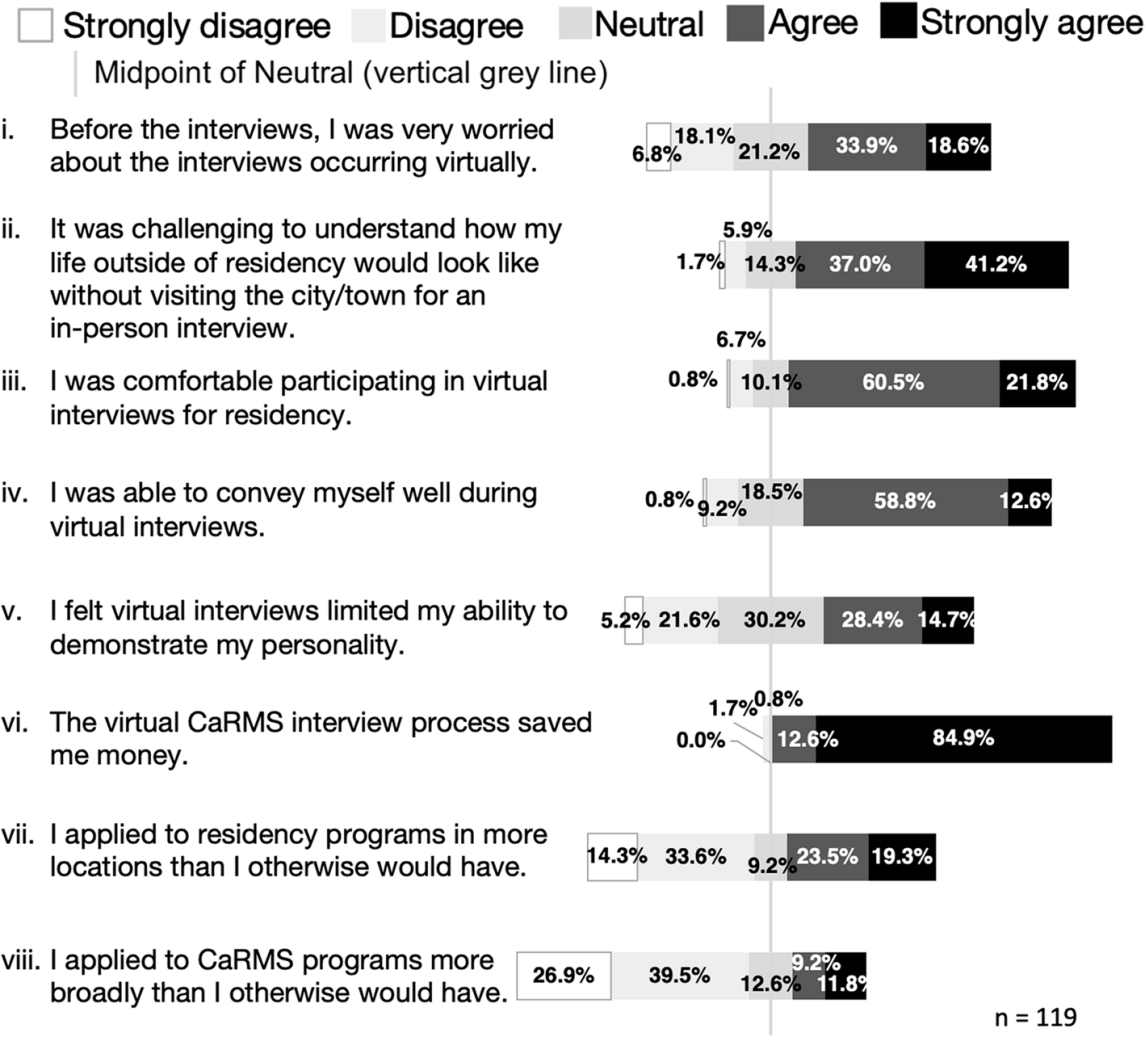
Residency applicants’ agreement to statements about the virtual CaRMS interview

Applicants’ perceptions of the virtual CaRMS interview format are detailed in Fig. 1.

Applicants ranked the following interview formats as most to least preferred: (1) Panel Interview, (2) Traditional 1-on-1 Interview, (3) Multiple Mini Interviews (MMI), and (4) Asynchronous recorded responses (in which applicants video record their responses to questions displayed on their screen in the absence of direct interaction with a real-time interviewer). Applicants’ reasons for preferring or not preferring different virtual interview formats are described in Table 2.

**Table 2:** Applicants’ Reasons for Preferring or Not Preferring Different Virtual Interview Formats

	**Themes**	**Applicant’s Quote**
**1. Panel Interviews**	*Advantages*	
	Build connections with multiple interviewers	Opportunity to engage with multiple different people creates more opportunities to connect and showcase myself
	Opportunities to get to know interviewers	Seemed the most interactive, got to know a few of the individuals from the school. Everything in general felt more personal
	Ability to showcase self	Felt it gave me the greatest chance to showcase my interpersonal skills by interacting with multiple interviewers in real-time
	*Disadvantages*	
	Felt uncoordinated	Panel interviews often felt uncoordinated and rushed
**2. Traditional 1-on-1 Interviews**	*Advantages*	
	Ability to build rapport	It allowed me a bit more time with the interviewer to establish rapport in programs where I could not do any electives in due to [the] COVID-19 [pandemic] and therefore could not get personally acquainted with
	More conversational	One-on-one interview provided more opportunity for conversation, especially compared to a panel interview where the interview platform (e.g. Zoom) often focuses on one person talking in a group and so makes it harder to have a natural conversation with more than one person
	Ability to showcase self	In traditional interviews I felt I got to showcase more of myself and my personality as well as learn more about the programs
	*Disadvantages*	
	Narrow evaluation	One on one is too small, narrowed view
**3. Multiple Mini Interviews (MMI)**	*Advantages*	
	Multiple fair first impressions	Ability to have "multiple chances" at a fair first impression—even if you did poorly on one MMI station, you have others to make up for it
	Interactions with multiple interviewers	Multiple opportunities to interact with program members, less impact if there was a mistake or tech issue
	*Disadvantages*	
	Brief interactions	In a time where we couldn't attend for visiting electives or have real face-time, I think the panel or one-on-one style interviews are very very very important to have your best shot at having your interviewers really get to know you. MMI would make an already distant process feel completely anonymous
	Difficult to execute virtually	MMI (with being pulled back and forth in breakout rooms), I could tolerate but did not prefer as it was a very jarring experience
**4. Asynchronous Interviews**	*Disadvantages—*No applicants described advantages of Asynchronous Interviews	
	Inability to showcase self and build connection	Recorded responses were far and away the least pleasant experience. I felt stunted, and as though I had no opportunity to engage with people and showcase my ability to connect
	Inability to get to know interviewers and program	Pre-recorded responses feel awful to do and don’t provide any feedback or chance to ask questions
	Negatively impact rank decision	One school did only asynchronous and it felt like they didn't care to meet me at all. That's the only program that's rank position was influenced by how they did interviews. It was just so cold and distant

Of the 109 applicants who reported using all the following videoconferencing platforms – Zoom, Skype, Cisco WebEx, and Microsoft Teams – most applicants preferred Zoom as the videoconferencing platform of choice (106/109, 97.2%). Based on written responses from applicants about the future use of videoconferencing platforms (Question 29), they preferred the Zoom platform as they were most familiar with that platform and felt that it was reliable.

### Interviewers’ perspectives

Most interviewers agreed (218/385, 56.6%) or strongly agreed (47/385, 12.2%) that they could easily evaluate applicants’ suitability for their program during the virtual interview. When comparing CaRMS interview formats, 193/378 (51.1%) interviewers felt that virtual and in-person interviews were equal in their ability to assess applicants, 177/378 (46.8%) interviewers felt that in-person interviews were better than virtual interviews, and only 8/378 (2.1%) interviewers stated that virtual interviews were better than in-person interviews for assessing applicant’s suitability for their program.

### Facilitators and barriers of the virtual CaRMS interview format

Facilitators of the virtual interview format include cost and time savings for both applicants and programs, ease of scheduling, reduced environmental impact, greater equity, less stress, greater reach and participation, and safety. Barriers of the virtual interview format include reduced informal conversations, limited ability for applicants to explore programs at different locations, limited ability for programs to connect with applicants and assess their interest, technological issues, concern for interview integrity, limited non-verbal communication, and reduced networking opportunities. A total of 70/388 (18.0%) interviewers and 38/123 (30.9%) applicants reported experiencing technological issues. Respondents reported internet connection problems as the most common technological issue (41/388 (10.6%) of interviewers and 17/123 (13.8%) of applicants). 56.3% (67/119) of applicants reported they accepted/participated in more interviews than they otherwise would have. Only 10.9% (42/384) of interviewers reported they had to participate in more interviews than previously. Themes representing the facilitators and barriers of the virtual CaRMS interviews are detailed in Table 3.

**Table 3:** Facilitators and Barriers of the Virtual CaRMS Interview

Facilitators			Barriers		
	**Interviewers** **N (%)**^**a**^	**Applicants** **N (%)**^**a**^		**Interviewers** **N (%)**^**a**^	**Applicants** **N (%)**^**a**^
Saved money	348 (90.6)	117 (98.3)	Reduced informal conversations	254 (66.1)	94 (79.0)
Saved applicants’ travel time	371 (96.6)	116 (97.5)	Could not get a feel for the applicants / program and city	169 (44.0)	Program: 78(65.5) City: 91(76.5)
Ease of scheduling	272 (70.8)	106 (89.1)	Concern about technological issues affecting the interview	105 (27.3)	29 (24.4)
Environmentally friendly	282 (73.4)	105 (88.2)	Could not communicate/ perform well virtually	51 (13.3)	28 (23.5)
**Themes**	**Quote(s)**		**Themes**	**Quote(s)**	
More equitable	Fewer barriers for those who struggle financially, single parents, etc. to make it more equitable for everyone participating. *-Applicant* Allowed candidates that might otherwise not be able to travel for either personal (parent, care giver) or financial reasons access to apply all programs. *-Interviewer*		Concern for interview integrity	I noticed some of the students looked like they were almost reading answers off of their screens for some of the basic questions, but it was important to know if they could also think on their feet and be more impromptu with their responses. *-Interviewer*	
Less stressful	From a mental health perspective, I think having virtual interviews was hugely beneficial, not having to coordinate travel, clothing, be away from home, etc.*-Applicant* Generally ran more efficiently and smoothly than in-person interviews. I truly hope we can maintain a virtual format in the future. *-Interviewer*		Difficult to discern genuine interest	Far less [interviews] were declined this year, interpreted to mean that many who have less legitimate interest in our program would still interview because it took far less time, money, and effort to join via zoom. *-Interviewer* Better sense of the candidates in terms of personality and fit when meeting in person.* -Interviewer*	
Greater reach of applicants	Allow opportunities to both save money and interview for more schools. *-Applicant* May attract candidates to interview who wouldn't have considered our program if they needed to travel here for a single interview. -*Interviewer*		Non-Verbals	Difficult to read individuals [interviewers], improvise and react in virtual environments. *-Applicants* In-person interviews permit evaluation of non-verbal cues, body language, eye contact. *-Interviewer*	
Greater interviewer participation	We had more interest in virtual interviews from faculty this year compared to when we were in-person surprisingly! Our rural teams often have to travel to the city to do interviews in winter, so they greatly appreciated the virtual space. *-Interviewer*		Reduced networking	Not only is it an enjoyable opportunity for candidates to relax and cut loose a little bit, [but] these interactions are important for framing oneself for future opportunities such as fellowships, job interviews, etc. if a good impression is made, etc.* -Interviewer*	
Safety	Less dangerous because usually interviews are in winter and transport during a storm can be dangerous*-Interviewer*		Less enjoyment	More pleasant to meet in person, not only for the applicants but also for the discussion between the members of the interview committee. *-Interviewer*	

^a^Of 384 interviewers and 119 applicants

### Respondents’ recommendations for improving the virtual CaRMS process

#### Recommendations regarding dissemination of information

Applicants (*n* = 38) and interviewers (*n* = 18) requested better opportunities for applicants to learn about programs outside of the virtual CaRMS interviews. Applicants (*n* = 10) recommended that virtual town hall sessions be mandated and recorded for later access. Applicants (*n* = 6) and interviewers (*n* = 4) recommended recruitment videos and online virtual tours to give applicants a better understanding of the program and what life is like in the city or town. Both applicants (*n* = 22) and interviewers (*n* = 14) recommended informal social events, offered prior to the interviews or during the interview period, for opportunities to connect with residents and staff, and develop genuine impressions of programs and culture. Some applicants (*n* = 6) reported preferences for small group or 1-on-1 socials, as it was difficult to learn the truth about programs in large virtual settings. Additionally, applicants (*n* = 11) recommended for detailed information to help prepare for the interviews, including the format of the interview, the videoconferencing platform used, and clarifications with respect to time zones.

#### Recommendations regarding the use of technology

Applicants (*n* = 14) reported stress during technological issues. Only a quarter of applicants (24.5%, 23/94) reported access to live support when a technological issue occurred. To reduce the impact of technological issues, interviewers (*n* = 17) and applicants (*n* = 2) recommended live tech supports, preferably provided nationally. Additionally, applicants (*n* = 15) and interviewers (*n* = 11) recommended a national standardized videoconferencing platform across all programs so that they can become familiar with the platform and thus minimize technological issues.

While most applicants (98.3%, 117/119) reported cost savings with respect to the virtual CaRMS interviews, they did comment on the additional costs for new hardware and for upgrading their internet connection. Applicants (*n* = 5) and interviewers (*n* = 7) recommended medical schools and/or residency programs offer applicants and interviewers additional resources, including space, equipment, and a reliable internet connection to help offset these additional costs, mitigate inequities experienced by applicants, and reduce the number of technological issues. Additionally, interviewers (*n* = 13) and applicants (*n* = 2) recommended the development of unified guidelines and training for interviewers to reduce bias during the virtual interviews, in attempts to minimize unfair assessments based on the quality of interview equipment, video background, and unexpected disturbances external to the applicant’s control.

Lastly, interviewers (*n* = 13) recommended development and use of a technology to prevent applicants from reading off their screens, since interviewers were not able to verify whether applicants were using prohibited aids. The use of a second camera, monitoring software and added distance between the applicant and their device was suggested.

#### Recommendations regarding the virtual interview format

Overall, most of the applicants (76.3%, 90/118) and over half of the interviewers (50.8%, 193/380) preferred a virtual over in-person CaRMS interview format to be used in the future. Some interviewers (*n* = 23) and applicants (*n* = 4) recommended the choice of either virtual or in-person interviews. However, the choice of interview format was argued against by some interviewers (*n* = 7) because of the potential for preferential bias for applicants with the resources to attend in-person interviews. Interviewers (*n* = 5) recommended for CaRMS to decide whether interviews should be made virtual or in-person, and the decision should be made unified across the country, or at least within each CaRMS discipline. One compromise suggested by some interviewers (*n* = 6) was the use of centralized in-person interviews for each discipline.

In summary of recommendations for previously discussed virtual interview formats, most applicants (*n* = 36) recommended panel interviews because of its conversational nature, ability to build connections with multiple interviewers, and chance to showcase their personal experiences. These merits were not possible with asynchronous interviews recommended against by applicants (*n* = 32).

## Discussion

In a web-based cross-sectional survey study of 2021 CaRMS R1 applicants and residency program interviewers, we identified a shared acceptance of the virtual interview format by both applicants and interviewers. We showed applicants’ preferences for virtual panel interview formats and identified facilitators and barriers of the virtual interview format. We enriched the quantitative results of our study by pairing them with analyzed, qualitative responses.

Most applicants and just over half of interviewers in our study preferred a virtual over in-person format for the CaRMS R1 interviews. Previously published studies reported similar preferences regarding virtual interviews among both fellows and program directors in internal medicine subspecialties [6], urologic oncology [4], and cardiothoracic programs [19]. Applicants and interviewers from our study described perceived advantages of virtual interviews that promote greater equity in the application process, including financial savings, time efficiency, and a greater reach and participation across applicants and interviewers. This is important given the recognized and unfulfilled need for greater equity in undergraduate and postgraduate medical education [20–22]. The continued use of the virtual interview format can help mitigate some of the inequities currently faced by residency applicants [23].

Despite most applicants’ preference to continue with the virtual format in future CaRMS R1 interview cycles, approximately half of interviewers (177/378, 46.8%) still felt that in-person interviews were better than virtual interviews for assessing applicants’ suitability for residency programs, and a quarter of applicants still preferred in-person over virtual interviews. These preferences may be explained by studies in the business literature and psychology literature comparing in-person and virtual interviews in the absence of factors external to the interview itself (eg. travel). These studies posit two relevant theories: [1] media richness [24]—the capacity of a communication medium to convey multiple verbal and nonverbal cues, allow for immediate feedback, use natural language, and provide a personal focus; and [2] social presence [25]—the capacity for interviewers and interviewees to experience and perceive the presence of one another within the communication medium. Studies of interviewers and interviewees assigned to different interview media described that in-person, virtual, and asynchronous interviews are associated with decreasing levels of media richness and social presence, respectively [26–30]. The type of interview medium has been shown to impact important objective measures of non-verbal communication, interview performance ratings, the ability for interviewees to make an impression of themselves, and perceptions of interview fairness [28, 29, 31–36]. Prior literature agrees with our findings, as respondents reported similar advantages of the in-person interviews, specifically the better use of non-verbal communication exchanges and better-perceived assessments by interviewers of applicants' motivation, commitment, and personality. Since clear differences exist between different interview formats, programs should attempt to use the same interview medium for all programs within the same discipline to promote fairness and equity during the interview process [28, 31, 35].

Our results show that about a third of applicants (38/123, 30.9%) experienced technological issues, and applicants perceived a negative impact on their interview performance as a result. A study by Fiechter et al. examined the impact of audiovisual technological problems on the assessments of job candidates in an experimental setting and demonstrated evidence for audiovisual (AV) quality bias [37]. Ratings of interview videos edited with simulated picture freezing, light-balance distortion, and a background static noise were compared to the ratings of original videos with maximum AV quality. The technological problems led to lower interview performance ratings, which was not relieved when assessors were told instructions to disregard the technological problems and not let video quality impact their assessments [37]. The AV quality bias has a troubling implication for the relatively high percentage of applicants who had experienced technological issues during the virtual CaRMS interviews, especially because it may place candidates with poor internet connection or unreliable devices at a disadvantage.

Resident interviewers were more likely to prefer in-person interviews compared to program directors and other faculty. Reduced informal conversations between applicants and programs was noted as a barrier of the virtual CaRMS interviews, as informal conversations could offer opportunities for interviewers to discern applicants’ personality. Since resident interviewers will be working closely with the matched applicants for the duration of their training, they may place greater emphasis on these informal conversations offered during in-person interviews that give genuine opportunities to discern applicants’ personality and their “fit” for the program.

The virtual interview format led to additional barriers including reduced informal conversations and difficulties with building connections, depending on the interview format. Prior studies made recommendations to minimize the barriers of technological media [29, 34]. Greater experience with using technology and greater affinity of interviewees for technology was associated with improved interview performance using virtual media [29]. CaRMS applicants should consider preparing for virtual CaRMS interviews using virtual technology to help optimize their performance, not only from a technological standpoint, but also in their perceived ability to make a natural impression of themselves during the interview [29, 34]. With the more frequent use of virtual meetings in undergraduate medical training since the pandemic, we anticipate that applicants will more easily accept virtual interviews as a method of communication in future CaRMS cycles. Additionally. medical schools should provide opportunities for applicants to practice virtual interviews with the specific platform used prior to the CaRMS interview.

Our results demonstrate that applicants prefer synchronous over asynchronous interviews for their conversational nature, opportunity to build connections with interviewers, and ability to showcase themselves. Previous studies indicate that the advantages of synchronous over asynchronous interviews include improved applicant motivation, performance, and ability to develop a positive impression during the interview [33]. The impression interviewers make during the interview plays a role in applicant recruitment and in their perception of the company [38], which is absent in interviews that solely use asynchronous media.

Based on the results of our study, we recommend moving future iterations of the CaRMS R1 interviews to a virtual format to make the interviews more equitable, to save money for the applicants, and to decrease the carbon footprint of the CaRMS interview tour [39]. Applicants reported challenges in learning about programs using the virtual interview format. Dissemination of information by residency programs can be improved using media preferred by applicants, including virtual townhall sessions, recruitments videos, and informal small group events. Moreover, visiting electives will provide applicants with adequate opportunities to learn about the programs while retaining all the benefits of the virtual format for the CaRMS interviews. Prior research has shown that visiting electives substantially impact the choice of specialty and location of future practice for applicants [1, 2, 40, 41].

### Limitations

Our study has several limitations. First, we attempted to obtain the emails of the appropriate medical school representatives for all Canadian medical schools but could only confirm email distribution to 5 Canadian medical schools, all that were English-speaking. We were not successful in obtaining the emails of appropriate medical school representatives for French-speaking schools and their emails were not made publicly available online. Second, we are not able to report an overall response rate for the applicants and the interviewers in our study as we do not know how many final-year medical students viewed the posts on social media and newsletters, and how many interviewers there were in total across all programs. The number of applicant respondents in our study may have been influenced by the timing of the survey distribution as it was distributed to the applicants after the end of the school year to comply with the ethical requirement to delay contact until after the R1 match date. Third, some R1 disciplines are underrepresented in our results, limiting specific conclusions with respect to those disciplines. There were no significant differences in applicants’ preferences for virtual or in-person interviews across disciplines with 10 or more applicants. This gives support for the generalizability of our results across disciplines. Additionally, the authors encourage the reader to review Table 1 to see if the results of our study are relevant to their specific discipline. Also, our results are subject to recall bias, as applicants completed the surveys 2–3 months after their CaRMS R1 virtual interviews, and self-report bias, as we did not have access to applicants’ CaRMS match data. Lastly, the applicants and interviewers in our study were asked to compare their perspectives on virtual and in-person interviews, but they may not have had any experience with in-person residency interviews. This may have biased their responses.

## Conclusion

The virtual format for the 2021 CaRMS R1 interviews was favourably perceived by applicants and interviewers. Continued use of the virtual format for future iterations of the CaRMS R1 interviews may not only save applicants’ time and money but may also promote greater equity in the residency application process.

## Supplementary Information

**Additional file 1.**  Applicant andInterviewer Survey.

## Data Availability

All data generated or analyzed during this study are included in this published article and its supplementary information files.

## Abbreviations

CaRMS: Canadian residency matching service
R1 match: Entry-level residency match
AFMC: The Association of Faculties of Medicine of Canada
MMI: Multiple Mini Interviews
