# Supplementary material for: A Canadian survey of residency applicants’ and interviewers’ perceptions of the 2021 CaRMS R1 virtual interviews
Source: BMC Med Educ. 2023 May 30;23:392. doi: 10.1186/s12909-023-04397-9 (PMC10226872; doi:10.1186/s12909-023-04397-9)
Supplement: Supplementary file 1 — Additional file 1. Applicant andInterviewer Survey. [file 12909_2023_4397_MOESM1_ESM.docx]

Additional file 1: Applicant and Interviewer Survey

Adaptive questioning is described by the blue boxes. The number of questionnaire items per page is described between ‘*Survey page break*.’ The questionnaire was distributed over 7 pages for applicants and 5 pages for interviewers. All questions were voluntary and non-mandatory. Respondents had the option to review and change their answers using a “Back” button.

Project Title: Applicant and Interviewer Perceptions of the CaRMS R1 Virtual interviews during the COVID-19 Pandemic.

Start of Block: Consent

Letter of Informed Consent. Survey respondents were informed of the research team members, the purpose of the study, length of time of the survey, and the data storage protocol. No incentives were offered.

- YES- I consent to participate in this study
- NO- I do not consent to participate in this study

Skip To: End of Survey If Queen's University HSREB Letter of Information-Consent Form = NO- I do not consent to participate in this study

***Survey page break***

**Section 1/5: Demographics**

1. Are you a:

- CaRMS applicant
- Program Director or Member of the CaRMS Selection Committee

2. Please create your unique personal code below - used to remove your responses upon withdrawal request

Last two letters of mother's maiden surname

________________________________________________

Last two digits of cell phone number

________________________________________________

End of Block: Consent

Start of Block: Applicant Survey Demographic Questions

3. How do you self-identify in terms of gender?

- Man
- Woman
- I do not identify within the gender binary
- I prefer not to answer

4. Do you identify as one or more of the following?

- Student with financial needs
- Student from a remote or underserved community
- None of the above
- I prefer not to answer

5. School of undergraduate medicine training:

- University of British Columbia
- University of Calgary
- University of Alberta
- University of Saskatchewan
- University of Manitoba
- Western University
- McMaster University
- University of Toronto
- Northern Ontario School of Medicine
- Queen’s University
- University of Ottawa
- McGill University
- Université de Montréal
- Université de Sherbrooke
- Université Laval
- Dalhousie University
- Memorial University of Newfoundland
- Other, please specify: _____________
- I prefer not to disclose

6. Residency program(s) applied to (please select all that apply from this list)

- Anatomical Pathology
- Anesthesiology
- Cardiac Surgery
- Dermatology
- Diagnostic Radiology
- Emergency Medicine
- Family Medicine
- General Pathology
- General Surgery
- Hematological Pathology
- Internal Medicine
- Medical Genetics and Genomics
- Medical microbiology
- Neurology
- Neurology – Pediatric
- Neuropathology
- Neurosurgery
- Nuclear Medicine
- Obstetrics and Gynecology
- Ophthalmology
- Orthopedic Surgery
- Otolaryngology – Head and Neck Surgery
- Pediatrics
- Physical Medicine & Rehabilitation
- Plastic Surgery
- Psychiatry
- Public Health and Preventative Medicine
- Radiation Oncology
- Urology
- Vascular Surgery

*Survey page break*

*Survey page break*

**Section 2/5: The CaRMS Pre-Interview Experience during the COVID-19 pandemic**

7. Which were helpful in providing information about the program, city, and culture of the institutions to which you applied? Please rank all that apply. (1 = most helpful)

- Virtual townhall
- Online videos created by the program
- Emailed information about the program, city, and culture
- Information on the CaRMS/AFMC websites
- Residency program website
- Residency program social media presence (e.g. Twitter/Facebook/Instagram)
- Informal discussions with residents/alumni outside of scheduled events
- Interview social (offered to candidates selected for the interview)
- Other (please specify): ________________________________________________

8. How did you prepare differently for virtual interviews compared to in-person interviews? (check all that apply)

- Optimized the remote physical space (1)
- Conducted a technology trial run (2)
- Practiced virtual interviews with friends or family (3)
- Completed a practice virtual interview session with faculty from my school
- Other (please specify): ______________________________________________

9. What steps did you take to optimize your virtual interview from a technology standpoint? (Select all that apply)

- Used a dedicated interview room on campus
- Moved to a location with reliable internet access
- Purchased new hardware (laptop/microphone/webcam/video lamp)
- Upgraded my internet connection
- Other (please specify):

________________________________________________

*Survey page break*

Section 3/5: The CaRMS Virtual Interview Format

[Statement] As a result of the COVID-19 pandemic, all R1 residency interviews were mandated to occur virtually across Canada. This is an unprecedented change that has never occurred in previous R1 CaRMS matches.

10. Which type of virtual interview format did you prefer? Please rank all that apply. (1 = most preferred)

- Panel Interviews
- Traditional one-on-one interviews
- Multiple mini interview (MMI) format
- Recorded responses for immediate upload
- Other (please specify): ________________________________________________

11. Why did you prefer your top-ranked interview format (as above)?

________________________________________________________________

________________________________________________________________

________________________________________________________________

________________________________________________________________

12. Please rank your preferred videoconferencing platform for the virtual interviews. (1 = most preferred)

- Zoom
- Skype
- Cisco WebEx
- Microsoft Teams
- Other (please specify):

________________________________________________

13. Did you experience technical issues during the interview?

- Yes
- No

Display Questions 14 if:

13. Did you experience technical issues during the interview = **Yes**

14. What technical issues did you experience? Please select all that apply.

- Loss or delay of internet connection
- The interviewer could not hear or see me
- I could not hear or see the interviewer
- Wrong interviewer/break out room assignments
- Hardware failure
- Issues with the videoconferencing program (e.g., Zoom)
- Other (Please specify): _________________

*Survey page break*

**Section 4/5: The CaRMS Virtual Interview Experience**

|  | Strongly disagree | Disagree | Neutral | Agree | Strongly agree |
| --- | --- | --- | --- | --- | --- |
| 15. Before the interviews, I was very worried about the interviews occurring virtually. |  |  |  |  |  |
| 16. It was challenging to understand how my life outside of residency would look like without visiting the city/town for an in-person interview. |  |  |  |  |  |
| 17. I was comfortable participating in virtual interviews for residency. |  |  |  |  |  |
| 18. I was able to convey myself well during virtual interviews. |  |  |  |  |  |
| 19. I felt virtual interviews limited my ability to demonstrate my personality. |  |  |  |  |  |

20. Please elaborate how you felt the virtual format impacted your interview performance.

________________________________________________________________

________________________________________________________________

________________________________________________________________

________________________________________________________________

________________________________________________________________

| 21. The virtual CaRMS interview process saved me money. |  |  |  |  |  |
| --- | --- | --- | --- | --- | --- |
| 22. I applied to residency programs in *more locations* than I otherwise would have. |  |  |  |  |  |
| 23. I applied to CaRMS programs more broadly (e.g., family medicine, general surgery) than I otherwise would have. |  |  |  |  |  |

24. How would you rate the virtual CaRMS interview process overall?

- Very poor
- Poor
- Satisfactory
- Good
- Excellent

*Survey page break*

**Section 5/5: Virtual CaRMS Interview General Feedback**

25. In your opinion, what were the strengths of the virtual CaRMS interview? Please select all that apply.

- Saved money
- Saved travel time
- Ease of scheduling
- Accepted/participated in more interviews than I otherwise would have
- More environmentally friendly
- Other (please specify): ________________________________________________
- Other (please specify): ________________________________________________

26. In your opinion, what were the weaknesses of the virtual CaRMS interview? Please select all that apply.

- Inability to have informal conversations with residents and staff
- Could not get a feel for the program
- Could not get a feel for the city
- Felt I could not perform well in the virtual format
- Concerns about technical issues affecting performance
- Other (please specify): ________________________________________________
- Other (please specify): ________________________________________________

27. In the future, do you think programs should conduct interviews virtually or in-person?

- Virtual
- In-person

28. What are your reasons for choosing a virtual or in-person interview?

________________________________________________________________

________________________________________________________________

________________________________________________________________

________________________________________________________________

________________________________________________________________

29. How could the virtual interview process for the R1 CaRMS Match be improved?

________________________________________________________________

________________________________________________________________

________________________________________________________________

________________________________________________________________

________________________________________________________________

30. Any other comments/concerns?

________________________________________________________________

________________________________________________________________

________________________________________________________________

________________________________________________________________

________________________________________________________________

End of Block: Applicant Survey Questions

Start of Block: Interviewer Questions

Display Questions 31-45 if:

Question 1. Are you a: = **Program Director or Member of the CaRMS Selection Committee**

**Section 1/4: Demographics**

31. I am:

- A Program Director
- Faculty member
- Resident
- Other (please specify):

________________________________________________

32. My residency program is:

- Anatomical pathology
- Anesthesiology
- Cardiac surgery
- Dermatology
- Diagnostic Radiology
- Emergency medicine
- Family medicine
- General Pathology
- General surgery
- Hematological Pathology
- Internal medicine
- Medical Genetics and Genomics
- Medical Microbiology
- Neurosurgery
- Neurology
- Neuropathology
- Nuclear medicine
- Obstetrics and gynecology
- Ophthalmology
- Orthopedic surgery
- Otolaryngology – Head and neck surgery
- Pediatrics
- Physical medicine and rehabilitation
- Plastic surgery
- Psychiatry
- Public health and preventive medicine
- Radiation Oncology
- Urology
- Vascular Surgery

*Survey page break*

**Section 2/4: Residency Program Information**

Display Question 33:

If Question 31: I am: = A Program Director

33. What medium(s) was employed to provide information about your residency program? Please select all that apply.

- Virtual townhall
- Online videos created by your program
- Emailed information about the program, city, and culture
- Information on the CaRMS/AFMC websites
- Residency program website
- Residency program social media presence (e.g., Twitter, Facebook, Instagram)
- Interview socials (offered to candidates who were selected for the interview)
- Other (please specify): ________________________________________________

*Survey page break*

**Section 3/4: The Virtual Interview Experience**

34. I was able to easily evaluate applicants' suitability for our program during the virtual interview.

- Strongly disagree
- Disagree
- Neutral
- Agree
- Strongly agree

35. Please compare your ability to assess applicants’ suitability for residency training in virtual interviews compared to in-person interviews.

- Virtual and in-person interviews were equal in their ability to assess applicants
- Virtual interviews were better to assess applicants than in-person interviews
- In-person interviews were better to assess applicants than virtual interviews

36. Please explain your above comparison of virtual to in-person interviews.

________________________________________________________________

________________________________________________________________

________________________________________________________________

________________________________________________________________

________________________________________________________________

37. Did you experience technical issues during the virtual interview(s)?

- Yes
- No

Display Question 38 if

Question 37: Did you experience technical issues during the interview(s) = Yes

38. What technical issues did you experience? Please select all that apply.

- Loss or delay of internet connection
- The interviewee could not hear or see me
- I could not hear or see the interviewee
- Wrong interviewee/break out room assignments
- Hardware failure
- Issues with the video conferencing program (e.g., Zoom)
- Other (Please specify): _________________

39. How would you rate the virtual CaRMS interview process overall?

- Very poor
- Poor
- Satisfactory
- Good
- Excellent

*Survey page break*

**Section 4/4: Virtual CaRMS Interview General Feedback**

40. In your opinion, what were the strengths of the virtual CaRMS interview? Please select all.

- Saved applicants’/program’s money
- Saved applicants’ travel time
- Ease of scheduling
- More environmentally friendly
- Other (please specify): ________________________________________________
- Other (please specify): ________________________________________________

41. In your opinion, what were the weaknesses of the virtual CaRMS interview? Please select all.

- Inability to have informal conversations with the applicants
- Could not get a feel for the applicants (e.g., genuine interest in the program/personality)
- Felt I could not communicate well virtually
- Concern about technical issues affecting applicants’ interview
- Had to participate in more interviews than previously
- Other (please specify): ________________________________________________
- Other (please specify): ________________________________________________

42. Given the opportunity next year, would you continue to conduct interviews virtually or return to in-person interviews?

- Virtual
- In-person

43. Please explain your rationale for your above choice of virtual or in-person interviews.

________________________________________________________________

________________________________________________________________

________________________________________________________________

________________________________________________________________

________________________________________________________________

44. How could the virtual CaRMS interviews be improved in the future?

________________________________________________________________

________________________________________________________________

________________________________________________________________

________________________________________________________________

________________________________________________________________

45. Any other comments or concerns?

________________________________________________________________

________________________________________________________________

________________________________________________________________

________________________________________________________________

________________________________________________________________

End of Block: Interviewer Questions
